# Gut microbiota- and probiotic-derived bioactive metabolites: mechanisms, evidence, and therapeutic potential in metabolic disorders

**DOI:** 10.3389/fcimb.2026.1953273

**Published:** 2026-10-12

**Authors:** Oghenerobor B. Akpor, Ayotunde O. Ajinde

**Affiliations:** 1 Department of Biological Sciences, Afe Babalola University, Ado-Ekiti, Ekiti State, Nigeria; 2 Department of Microbiology, Federal University of Agriculture and Development Studies, Iragbiji, Nigeria

**Keywords:** gut microbiota, metabolic diseases, non-communicable diseases, postbiotics, probiotic strains

## Abstract

The gut microbiota is a taxonomically, genetically, and metabolically diverse and dynamic community of different microbial groups, including bacteria, archaea, fungi, and viruses, which are either in mutualistic or commensal relationships with their host, performing important metabolic regulation functions that bring a range of health benefits through the action of a wide range of bioactive compounds. These include short-chain fatty acids, secondary bile acids, exopolysaccharides, conjugated linoleic acids, tryptophan and indole derivatives, bacteriocins and antimicrobial peptides, and vitamins. Their health benefits include intestinal epithelial integrity, immunomodulation, metabolism, energy homeostasis, and host-gut microbiome interactions. A shift in the compositional and functional balance of the gut microbiota has been strongly linked to various metabolic disorders, including obesity, type 2 diabetes mellitus, metabolic syndrome, and non-alcoholic fatty liver disease. The use of these probiotic-derived metabolites as an alternative or complement to standard therapeutic procedures is gaining traction within the scientific community because of potential benefits such as improved stability, standardized dosing, minimal risks of genetic mutability through gene transfer, and pathogenicity. In this narrative review, we searched popular databases such as PubMed and Google Scholar for relevant materials, with emphasis on quality and rigor. This review discusses the current state of knowledge concerning the principal groups of gut microbiota- and probiotic-derived metabolites, their biosynthetic pathways, mechanisms of action, and origin. It presents a synthesized knowledge of the potential therapeutic benefits of these bioactive molecules in metabolic disorders. An attempt is made to critically evaluate their therapeutic benefits from available evidence. The review presents an interspersed discussion of the key constraints limiting the applicability of these metabolites in clinical metabolic therapy. It ends by highlighting key future research areas necessary for the eventual applicability of these metabolites as effective agents against metabolic diseases.

## Graphical Abstract

**Figure:**
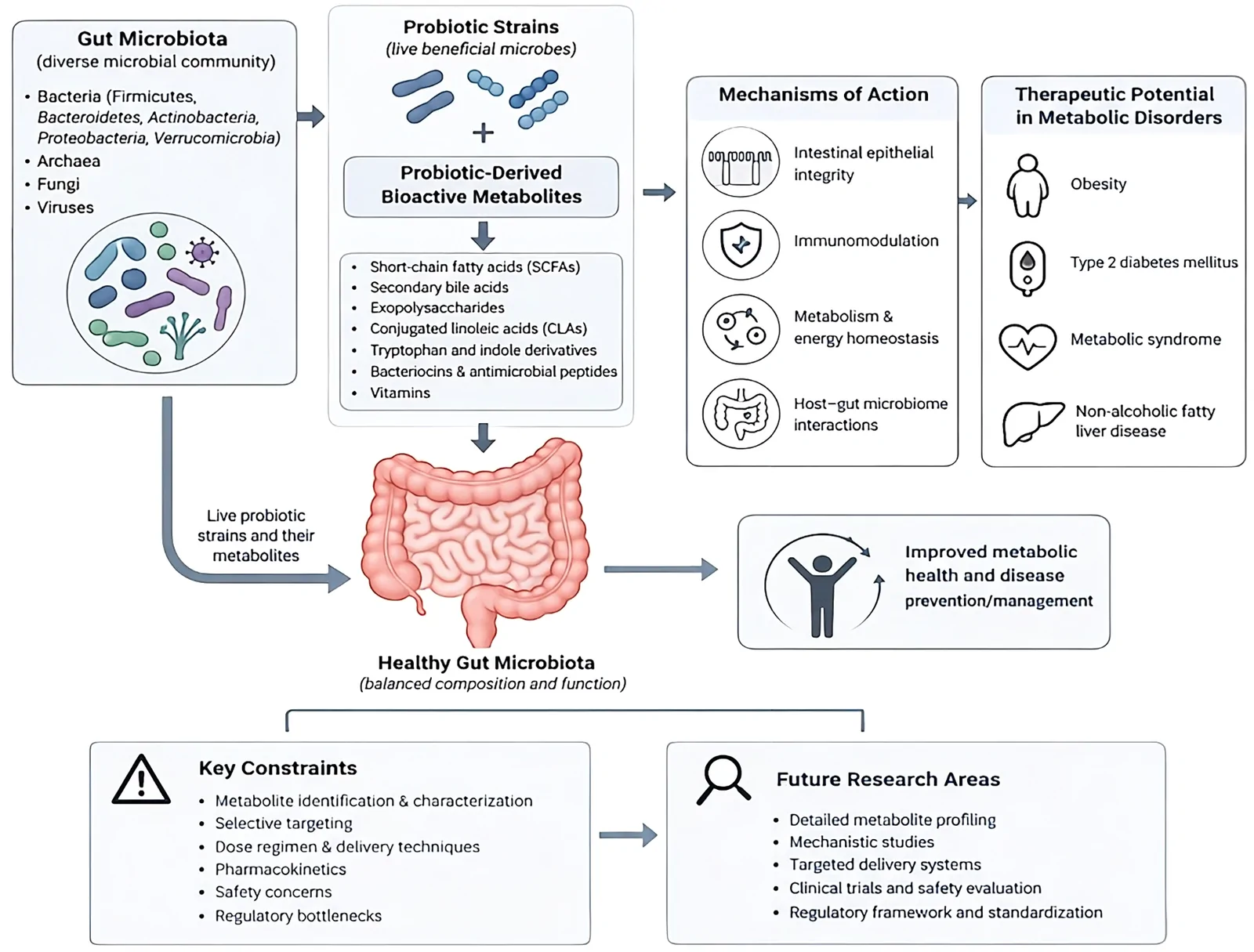
Overview of probiotic strains and bioactive metabolites, their key mechanisms of action, therapeutic targets, operational constraints, and future research directions.

## 1 Introduction

The human gut microbiota is a complex and intricate community of diverse microbial groups in the human digestive tract, existing in mutualistic or commensal relationships with the host. Over 2000 species, distributed into 12 phyla, are members of the human gut microbiome (Thursby and Juge, 2017), with bacteria forming the overwhelming majority. The most common phyla are Firmicutes, Bacteroidetes, Actinobacteria, Proteobacteria, and Verrucomicrobia (Rinninella et al., 2019), with anaerobes being the most predominant bacterial group, representing 99% of total bacterial populations (Thursby and Juge, 2017).

This dynamic community consists not only of bacteria but also of several other simple unicellular organisms and, less importantly, fungi, archaea, and viruses. In terms of pure mass, the number of bacterial cells is roughly equal to that of human cells in the body (Sender et al., 2016). This vast assemblage of microbially diverse species contains up to 3.3 million genes, far more than the approximately 23, 000 genes in the human genome, highlighting their scale and potential importance for host health (Qin et al., 2010). While there are a few microorganisms inhabiting the stomach and intestine, the colon hosts the largest population of these living organisms.

Broadly speaking, it is possible to classify host organisms based on the composition of gut microbiota into enterotypic groups (Mobeen et al., 2018). The term gut microbiota refers to the totality of microbial species and their genomes and functional attributes (Ursell et al., 2012). Their primary functions include regulation of the internal host environment and modulation of host immune responses (Rooks and Garrett, 2016). A host of factors influence these host–microbiome interactions, making them both dynamic and bidirectional. Through diet composition, genetic makeup, medications, and gut environmental conditions, the host modulates microbial communities in structure, composition, and function. In turn, structural moieties and microbial secretions serve as signaling molecules to epithelial, immune, and neuroendocrine systems in the regulation of body metabolism.

The concept of probiotics as a healthy choice for intestinal health and longevity can be attributed to the Nobel Prize winner in Medicine and Physiology, Metchnikoff (1845–1916), the Russian zoologist (Smug et al., 2014). Over time, various definitions have been put forward, first by Fuller (1989), and later by Schrezenmeir and De Vrese (2001) and FAO/WHO (2001). A synthesis of these definitions produces one that defines probiotics as live microbial species with the capacity to produce positive health benefits when consumed in sufficient amounts. The regulatory framework around probiotics seems to be evolving and in a state of flux (Cordaillat-Simmons et al., 2020)

A good candidate for probiotic effects should be non-pathogenic, non-toxic, non-mutagenic, non-carcinogenic; it must be capable of gastric and intestinal transit without loss of functionality; it must be genetically stable with no plasmid transfer capability; it must be amenable to food manufacturing, processing, transport, storage conditions; it must be capable of effective colonization of the intestine, and it must be capable of producing its beneficial functional effects at a satisfactory level upon consumption (Schiffrin and Blum, 2001). This definition presumes that such species produce functional effects *in vivo*, in animal studies, and in human trials. The vast majority of known probiotic organisms belong to one of these genera: *Bacillus*, *Lactobacillus*, *Saccharomyces*, and *Bifidobacterium* (Bermudez-Brito et al., 2012; Bajaj et al., 2015).

Despite being a highly heterogeneous environment in terms of taxonomic, genetic, and functional traits, collectively they exert profound physiological effects on host well-being through the fermentation of indigestible dietary materials to produce energy molecules such as short-chain fatty acids; biosynthesis of bioactive molecules, vitamins, and secondary metabolites; contribution to intestinal epithelial integrity; modulatory effects on immune cells; and biodegradation of recalcitrant molecules. Moreover, their activities in the gut lead to the competitive exclusion of pathogenic species.

Gut microbiota development in humans begins at infancy and continues throughout life, with different parts of the gut showing different structural and functional microbial compositions. Stability is finally established at a certain age, after a period of rapid transformation starting from childhood (Guittar et al., 2019). An increase in microbial diversity is usually, but not always, linked to better health outcomes, although in some cases it can be a characteristic feature of certain metabolic diseases (Sommer et al., 2017; Larsen and Claassen, 2018). Several factors can modulate the composition of gut microbiota, including diet, mode of delivery and feeding, age, host genotype, sex, body mass index, and antibiotic use, as highlighted by Bander et al. (2020). Together, these factors influence colonization and the eventual functional efficacy of the bacterial strain. The important beneficial effects of probiotics have provided impetus for the development of probiotic food products.

While effective colonization is an important factor for their functionality, the consensus is that they produce bioactive molecules and structural components through which they exert their beneficial effects. There is a marked degree of chemical diversity among these bioactive metabolites and microbial products, collectively called postbiotics. The International Scientific Association for Probiotics and Prebiotics (ISAPP) defines postbiotics as consisting of dead microorganisms and/or their structural or cellular components that provide positive health benefits (Salminen et al., 2021). The definition excludes non-cellular materials, macromolecules (e.g., bacteriocins, peptides, exopolysaccharides (EPS), enzymes), purified microbial metabolites such as indole, microbial peptides, lipids, metabolic products derived from indole and tryptophan, vitamins, EPS, secondary bile acids, and short-chain fatty acids (SCFAs) (acetate, propionate, and butyrate). These explicit exclusions by ISAPP serve to correct literature inconsistencies common in older sources. Other related but conceptually confusing terms include prebiotics, which are non-digestible, fermentable substrates, typically but not limited to carbohydrates, found in foods that promote the proliferation of gut microbiota (Lockyer and Stanner, 2019; Rastall et al., 2022), and synbiotics, which are a collective term for both probiotics and prebiotics (Chauhan and Chorawala, 2012; Pandey et al., 2015) (**Table 1**).

**Table 1:** Terminology and product taxonomy to avoid terminological confusion.

Product class	Definition	Examples	Important note
Purified microbial metabolites	Small molecules with definite and known chemical structures	Indoles, SCFAs, secondary bile acids, vitamins	Explicitly not postbiotics unless there is a cellular component or biomass (Salminen et al., 2021)
Secreted microbial products	Molecules produced by microorganisms and released into the environment	Cell-free proteins, peptides, EPS, enzymes, bacteriocins	Explicitly not postbiotics without a cellular component or biomass (Salminen et al., 2021)
Postbiotics	A preparation of inanimate microorganisms and/or their components that confers a health benefit on the host.	Non-living cells, cell wall, membrane proteins, microorganisms, or cellular components	The presence of associated microbial metabolites is not a defining characteristic (Salminen et al., 2021)
Live probiotics (microbial preparations)	Living microorganisms administered in sufficient dose	Mostly live bacteria and yeasts	Live, viable microbial cells with proven benefits when taken in adequate amounts)
Prebiotics	Non-living dietary components with selective stimulatory effects on specific beneficial gut microbiota	Oats, inulin, garlic, and onions	Actually, it is a contradiction in terms since they are non-living food components. They serve to nourish specific beneficial gut microbiota
Synbiotics	A food or food supplement that combines both live probiotics and prebiotics into one single formula	Live microorganism (eg., bacteria or yeast) + non-digestible food components such as fibers, inulin or fructooligosaccharides	Proven beneficial effects are a defining characteristic (Salminen et al., 2021)

Non-communicable diseases (NCDs) are a growing global health menace worldwide (Nugent and Fottrell, 2019). This is partly due to the increasing incidence of metabolic diseases (Sethi and Hotamisligil, 2021). Proper functioning of the gut microbiota is dependent on the stability of a delicate balance. Disruptions to this delicate balance have been linked to metabolic diseases (Zhang et al., 2015; Stewart et al., 2016; Zou et al., 2018). These metabolic diseases, which include obesity, type 2 diabetes mellitus (T2DM), metabolic syndrome, and metabolic dysfunction-associated steatotic liver disease (MASLD), are a growing global health menace, affecting millions and elevating cardiovascular and cancer risks.

These conditions involve metabolic impairments and affect insulin sensitivity, glucose and lipid metabolism, immunoinflammatory cells, and cytokines (Sethi and Hotamisligil, 2021). They are common predisposing factors for deformities, cancer, or even death. Common functional bodily changes caused by these disorders and diseases include inflammation, dyslipidemia, insulin resistance, and energy imbalance. These pathophysiological changes are connected to changes in the structure, composition, and functionality of the gut microbiota. For instance, pathophysiological changes include reduced intestinal barrier function, causing an inflammatory response due to the movement of microbial lipopolysaccharide, and alterations in bile acid metabolism and SCFA profiles. This condition results in fat tissue inflammation, alterations in lipid metabolism, and host appetite, as a result of changes in microbial metabolites. There is a growing body of evidence to suggest that the positive modulatory effects of probiotics or their metabolites on the compositional structure of the gut microbial metabolome can lead to improved metabolic outcomes.

There is a vast array of gut microbiota-derived metabolites that have shown promising therapeutic potential against various metabolic conditions. The attractiveness of directly using these metabolites over live probiotic preparations potentially includes highly selective action, ease of preparation, testing, and analysis, precise dosing, minimal risks of genetic modification through gene transfer, and reduced risk of opportunistic infections. However, the full realization of the potential of these bioactive metabolites remains at a nascent stage. There is a need for definitive chemical identification and quantification of these metabolites, the elucidation of their molecular and systemic mechanisms of action, and the impact of host factors on effectiveness. There is also a need for thorough clinical evaluation of their dosage, optimal delivery methods, safety, and long-term health effects.

This review presents a synthesis of contemporary information on medically relevant, major classes of gut microbiota-derived microbial metabolites for metabolic disorders. It starts with a concise summary of the major classes of gut microbiota-derived microbial metabolites and their biochemical origins. It examines evidential information from animal models and human studies for the effectiveness of these metabolites in metabolic functions. Then it dives into a presentation of the different therapeutic approaches, such as probiotic-mediated secretion of active metabolites through engineered delivery, direct supplementation, and combined strategies that include prebiotic use or established protocols. Lastly, it discusses current constraints affecting translational use of these metabolites to finished, commercial, and safe products for human use, and highlights priority areas of future research endeavors.

## 2 Literature search strategy

This narrative review was conducted through a structured literature search of PubMed and Google Scholar. Search covered literature published from [YEAR] to [MONTH/YEAR], with additional relevant earlier publications included where necessary to provide foundational information on established concepts. Search terms were developed around four major concepts: gut microbiota/probiotics, microbial metabolites/postbiotics, metabolic disorders, and mechanisms of action. Representative search combinations included (“gut microbiota” OR probiotic* OR “probiotic-derived”) AND (metabolite* OR postbiotic* OR “microbial metabolite*”) AND (“short-chain fatty acid*” OR “bile acid*” OR bacteriocin* OR exopolysaccharide* OR indole* OR tryptophan OR “conjugated linoleic acid” OR vitamin* OR GABA) AND (obesity OR “type 2 diabetes” OR “metabolic syndrome” OR MASLD OR NAFLD). Priority was given to peer-reviewed original research, clinical trials, systematic reviews, meta-analyses and authoritative consensus documents. Studies were considered relevant when they addressed production, characterization, mechanisms, biological effects, therapeutic potential, safety, pharmacokinetics, formulation or delivery of microbial metabolites relevant to metabolic health. Publications that were unrelated to metabolic outcomes, lacked substantive information on the metabolites under consideration, or were limited to non-relevant microbial or host systems were excluded. Reference lists of key articles were also examined to identify relevant additional studies.

## 3 Gut probiotics and production of bioactive metabolites

Gut probiotics are indicated to play a vital role in the maintenance of host health through the production of a variety of bioactive metabolites that are produced during the biotransformation and fermentation of dietary and endogenous substrates. These metabolites include SCFAs, bacteriocins, amino acid derivatives, vitamins, and secondary bile acids, and they serve as important mediators of host–microbe interactions through the regulation of intestinal barrier integrity, immune function, inflammation, and metabolic homeostasis (Sanders et al., 2019). Several factors can influence the quantity and composition of these metabolites, including probiotic species, diet, host genetics, gut environmental conditions, and lifestyle (Koh, 2016; Sanders et al., 2019).

### 3.1 Major gut probiotic microorganisms

Gut probiotics are live microbial preparations with the capability to produce specific health benefits when administered in sufficient dosage. Many probiotic microorganisms naturally inhabit the gastrointestinal tract where they interact with resident microbiota, epithelial cells, and immune components. The majority belong to the phyla Firmicutes and Actinobacteria, although certain yeasts have also demonstrated probiotic properties (Sanders et al., 2019). While probiotic species are generally regarded as Generally Recognized As Safe (GRAS) or possess Qualified Presumption of Safety (QPS) status because of their long history of safe use in foods (Hill et al., 2014), safety cannot be inferred broadly based only on genus- or species-level identity or from historical food use alone (Colautti et al., 2022; da Silva et al., 2024; Churin et al., 2026). Their safety must be decided on a case-by-case basis, and this varies with jurisdiction and intended use. It is therefore necessary to establish an acceptable probiotic safety level through the assessment of pathogenicity and virulence factors, gene stability, gene transfer potential, antimicrobial-resistance determinants, toxic metabolites, product quality, and intended use before the clinical trial phase (Wang et al., 2021; Roe et al., 2022; Liang et al., 2024; Almagambetov, et al., 2026).

Major probiotic genera are *Lactobacillus* (recently reclassified into multiple genera such as *Lactobacillus*, *Limosilactobacillus*, *Lactiplantibacillus*, *Lacticaseibacillus*), *Bifidobacterium*, *Saccharomyces boulardii*, and other emerging probiotic species. Among the probiotic genera, *Lactobacillus* is traditionally the largest group. They are Gram-positive, facultative anaerobic rods and produce lactic acid as the major fermentation end-product. They are known to produce numerous bioactive metabolites, such as lactic acid, SCFAs, EPS, bacteriocins, and gamma-aminobutyric acid (GABA). In addition, they contribute to the maintenance of intestinal pH, inhibition of pathogens, and enhancement of epithelial barrier integrity (O'Callaghan and van Sinderen, 2016).

*Bifidobacterium* are dominant colonizers of the infant intestine. They are Gram-positive anaerobic bacteria belonging to Actinobacteria. They are efficient degraders of human milk oligosaccharides and dietary fibers. They are major producers of acetate, lactate, folate, conjugated linoleic acid (produced by certain strains), and promote immune maturation and cross-feeding interactions that stimulate butyrate-producing bacteria (Arboleya et al., 2016).

*Lacticaseibacillus*, *Limosilactobacillus* and *Lactiplantibacillus* were previously included within *Lactobacillus*. Examples include *Lacticaseibacillus rhamnosus, Lacticaseibacillus casei, Lactiplantibacillus plantarum*, and *Limosilactobacillus reuteri.* These species are important because they produce reuterin, bacteriocins, GABA, antioxidant peptides, and SCFAs. These metabolites regulate intestinal inflammation, glucose metabolism, and lipid homeostasis (Zheng et al., 2020).

*Saccharomyces boulardii* is a probiotic yeast that is indicated to show resistance to many antibacterial antibiotics. The yeast is implicated in enzyme production and anti-inflammatory factors. It also neutralizes bacterial toxins and restores microbial balance during antibiotic-associated diarrhea (McFarland, 2010). Other emerging probiotic species, such as *Akkermansia muciniphila, Faecalibacterium prausnitzii, Clostridium butyricum*, and *Propionibacterium freudenreichii* have been reported in the literature. These species are reported to produce high amounts of butyrate, propionate, extracellular proteins, and indole derivatives that improve metabolic homeostasis (Depommier et al., 2019).

### 3.2 Ecological roles within the gut

Gut probiotics contribute to host physiology through several ecological functions. They maintain colonization resistance by occupying ecological niches, competing with pathogens for nutrients, producing antimicrobial compounds, and reducing pathogen adhesion to intestinal epithelial cells. Some examples of antimicrobial substances include bacteriocins, organic acids, hydrogen peroxide, and reuterin.

Probiotics maintain intestinal barrier function through the stimulation of mucin secretion, enhancement of tight junction proteins, reduction of intestinal permeability, and improvement of epithelial regeneration. This action is indicated to limit metabolic endotoxemia associated with obesity and diabetes (Cani, 2018). With respect to immune regulation, probiotics regulate both innate and adaptive immunity through increase in regulatory T cells, promoting IL-10 production, suppression of TNF-α, IL-6, and IL-1β (Hemarajata and Versalovic, 2013).

In performing metabolic functions, gut probiotics produce numerous metabolites such as acetate, propionate, butyrate, indoles, conjugated fatty acids, vitamins, polyamines and neurotransmitters. These metabolites are indicated to influence insulin sensitivity, appetite regulation, lipid metabolism, hepatic gluconeogenesis, and inflammation (Postler and Ghosh, 2017).

For colonization and survivability, successful probiotic function depends on survival throughout the gastrointestinal tract. During colonization, probiotics encounter gastric pH values between 1.5 and 3.5. Some of the survival mechanisms include production of proton pumps, acid shock proteins, membrane lipid remodeling, and production of protective proteins. Bile salt resistance is another survivability strategy. Since bile salts damage bacterial membranes, many probiotics express bile salt hydrolase (BSH), stimulate membrane transporters and stress response proteins, which help to improve persistence within the small intestine (Govaert et al., 2024).

Any successful colonization by a probiotic is dependent on mucus-binding proteins, pili, surface-layer proteins, and EPS. Adhesion enhances prolonged metabolite production and host interaction (O'Callaghan and van Sinderen, 2016).

For competitive fitness, long-term persistence depends on nutrient acquisition, quorum sensing, biofilm formation, cross-feeding interactions, and resistance to host defenses. Also, probiotics colonize through cross-feeding mechanisms. Many probiotic metabolites become substrates for secondary fermenters. This metabolic cooperation greatly increases production of health-promoting SCFAs (Rivière et al., 2016).

### 3.3 Factors influencing metabolite production

Several factors, such as diet, presence of polyphenols, protein intake, fat intake, host genetics, gut environmental condition, antibiotics and lifestyle factors, physical activity, stress and sleep are known to influence metabolite production by probiotics. Diet is considered the strongest determinant of microbial metabolite production. Fermentable fibers, such as inulin, resistant starch, pectin, arabinoxylans, and fructooligosaccharides (FOS) are reported to increase production of acetate, propionate, and butyrate (Makki et al., 2018).

Substances, such as polyphenols undergo microbial biotransformation into bioactive metabolites, which include urolithins, phenolic acids and flavonoid derivatives. These are indicated to exhibit antioxidant activity, anti-inflammatory activity, and insulin-sensitizing effects. Protein fermentation generates beneficial and potentially harmful compounds. Some beneficial compounds include indole derivatives and tryptophan metabolites while the potentially harmful ones include ammonia, hydrogen sulfide, p-cresol and branched-chain fatty acids. Excessive protein fermentation may promote intestinal inflammation (Windey et al., 2012).

Also, high-fat diets could lead to reduced beneficial probiotic populations, decreased butyrate production, increased endotoxin-producing bacteria, and impaired gut barrier integrity (Cani, 2018). Host genetics is indicated to influence mucin glycosylation, immune signaling, antimicrobial peptide secretion, intestinal transit, and nutrient availability. These factors alter microbial community composition and metabolite production (Goodrich et al., 2016).

In the case of gut environmental conditions, the important environmental variables include pH, oxygen tension, redox potential, substrate availability, intestinal transit time and mucus composition. Anaerobic conditions particularly favor SCFA synthesis. Also, antibiotic exposure is reported to lead to reduced microbial diversity, suppressed metabolite-producing bacteria, decreased SCFA production and delayed recovery of metabolic functions.

Poor sleep quality has been associated with microbial dysbiosis, impaired SCFA production, and metabolic dysfunction (Dethlefsen and Relman, 2011). Physical activity, such as exercise, is associated with increased bacterial diversity and possibly SCFA concentrations (Clarke et al., 2014).

### 3.4 Biosynthetic pathways of probiotic metabolites

Fermentation is the primary mechanism through which probiotics generate bioactive metabolites. The major end-products of fermentation include acetate, propionate, butyrate, lactate, ethanol, hydrogen, and carbon dioxide.

Gut probiotics employ a diversity of metabolic pathways for the conversion of dietary substrates into bioactive metabolites that influence host physiology. Through metabolism of carbohydrate, amino acid, and lipid metabolism, these microbes can produce compounds that regulate metabolism, immune function, intestinal barrier integrity, and gut–brain communication.

Generally, glucose is metabolized via the Embden–Meyerhof–Parnas (EMP) glycolytic pathway to produce pyruvate using a homofermentative pathway. The pyruvate is subsequently reduced to lactate as the primary end-product. In many lactobacilli species, the EMP pathway is predominant and is characterized by the efficient conversion of carbohydrates into lactic acid with minimal production of other metabolites. Apart from the homofermentative pathway, glucose can also be metabolized using the heterofermentative pathway. The heterofermentative pathway for glucose metabolism utilizes the pentose phosphate/phosphoketolase pathway. In this pathway, glucose is converted into lactate in addition to other fermentation products such as acetate, ethanol, and carbon dioxide (CO_2_) through this route. The diversity in the physiological functions of heterofermentative probiotic bacteria within the gastrointestinal tract is attributed to the production of multiple metabolites via the heterofermentative pathway.

In addition to glucose, gut probiotics are reported to possess the ability to metabolize several dietary amino acids for the generation of bioactive compounds with important physiological functions. As an example, during the metabolism of tryptophan, metabolites such as indole-3-lactic acid, indole-3-acetic acid, and indole-3-propionic acid are produced. It is indicated that enhanced intestinal barrier integrity, stable immune homeostasis, and regulation of mucosal immune responses are due to AhR, which is activated by the presence of indole derivatives. In addition, the metabolism of glutamate into GABA has been reported in some probiotic strains. GABA is an important neurotransmitter that is important in glucose homeostasis, inflammatory response modulation, and facilitation of communication along the gut–brain axis. Furthermore, histamine is produced during the metabolism of histidine. Histamine is known to affect inflammatory processes through interactions with different histamine receptor subtypes. Histamine may either promote or suppress immune responses, depending on the activated receptor, thus contributing to immune regulation within the intestinal environment (Roager and Licht, 2018).

Metabolite production by probiotic microbes can also be carried out through lipid metabolism. Probiotic microorganisms participate in lipid metabolism through the production of several biologically active lipid-derived metabolites, such as linoleic acid (CLA), conjugated fatty acids, secondary bile acids, and sphingolipids. Also, several probiotic bacteria are reported to possess bile salt hydrolase (BSH) activity, which modifies the composition of a host bile acid pool. Bile salt hydrolases are known to activate the nuclear farnesoid X receptor (FXR) and the membrane-bound protein-coupled bile acid receptor, Takeda G-protein receptor 5 (TGR5). The regulation of lipid metabolism, glucose metabolism, and energy expenditure is carried out through the activation of these signaling pathways, thus contributing to improved metabolic homeostasis (Jia et al., 2018).

The fermentation of complex dietary carbohydrates is one of the most important metabolic activities of gut probiotics. In the upper gastrointestinal tract, dietary polysaccharides that escape digestion are first hydrolyzed into monosaccharides, which then enter glycolysis to produce pyruvate. The pyruvate is subsequently fermented into SCFAs, principally acetate, propionate, and butyrate. These SCFAs serve as key signaling molecules that regulate host metabolic functions. Mechanistically, SCFAs activate the G protein-coupled receptors GPR41 and GPR43, inhibit histone deacetylases, stimulate glucagon-like peptide-1 (GLP-1) secretion, improve insulin sensitivity, suppress inflammatory responses, and regulate hepatic gluconeogenesis. Through these mechanisms, SCFAs play a central role in maintaining metabolic homeostasis and reducing the risk of metabolic disorders (Koh et al., 2016).

## 4 Major gut microbiota- and probiotic‐derived bioactive metabolites

These diverse groups of microbial bioactive metabolites produce functional effects on host health in numerous ways. Presented below are the major groupings of probiotic‐derived bioactive molecules from representative probiotic genera (**Table 2**).

**Table 2:** Summary of the major metabolite classes.

Class	Examples	Producing species	Biosynthetic pathways	Target molecules/receptor	Physiological effects
SCFAs	Acetate, propionate, butyrate	*Lactobacillus* acidophilus, Lactiplantibacillus *plantarum, Bifidobacterium adolescentis*	Carbohydrate fermentation	GPR41, GPR43, HDACs	Modulates lipid metabolism; energy source for colonocytes (butyrate); gut barrier integrity; controls inflammation
Secondary bile acids	DCA, LCA	*Bifidobacterium*, *Lactobacillus*, *Bacteroides*. *Clostridium* and *Eubacterium*	Deconjugation and 7α/β‐dehydroxylation reactions	FXR, TGR5	modulates glucose and lipid metabolism; boosts energy expenditure; regulates bile acid pool; reduces systemic inflammation
Bacteriocin and antimicrobial peptides	Nisin, Pediocin, Sakacin, Lacticin	*Lactococcus lactis, Pediococcus acidilactici*	Ribosomal synthesis and post-translational modifications	Cell wall biosynthesis; cell membrane	Competitive inhibition of pathogenic species; reduces inflammation; indirectly regulates metabolism
Tryptophan and indole derivatives	Indole-3-acetic acid (IAA), Indole-3-aldehyde (IAld), Indole-3-propionic acid (IPA),	*Lactobacillus reuteri*, *Clostridium sporogenes*, *Bifidobacterium infantis*	Tryptophan metabolism	AhR, PXR	Enhances mucosal immunity; protects intestinal barrier; anti-inflammation; microbial communication
Conjugated Linoleic Acids (CLAs)	*cis*-9, *trans*-11 CLA; *trans*-10, *cis*-12 CLA	*Lactiplantibacillus plantarum*, *Bifidobacterium breve*	isomerisation of linoleic acid	PPAR-alpha, PPAR-gamma	Protect intestinal integrity; regulates glucose and fat metabolism; stimulates fat oxidation; modulates inflammation;
Vitamins (B-Complex & K)	B9, B2, B12; K2	*Bifidobacterium adolescentis, Lactococcus lactis, L. reuteri*	De novo biosynthesis	Enzyme cofactors	DNA synthesis and repair; antioxidant activity; energy metabolism
Neuroactive metabolites	GABA, serotonin	*Lactobacillus brevis*, *L. plantarum*, *Bifidobacterium dentium*	Glutamate decarboxylation	GABA receptors, vagus nerve afferents	Modulates gut-brain communication; slows bowel movement; improves appetite signalling;

### 4.1 Short-chain fatty acids

Humans lack the full assortment of enzymes for complete carbohydrate digestion. Probiotic strains inhabiting the colon have the needed enzymatic capabilities for the anaerobic bioconversion of these complex carbohydrates into SCFAs, such as butyrate, acetate, and propionate, which can be subsequently absorbed by colonocytes (Markowiak and Śliżewska, 2017). SCFAs provide 60–70% of the energy requirements of colonocytes and between 5 and 15% of total energy needs in humans (Brahe et al., 2013).

Acetate, which is produced by a diverse group of fermentative probiotic bacterial species, such as Firmicutes (*Lactobacillus acidophilus* and *Lactiplantibacillus plantarum*) and Actinobacteria (*Bifidobacterium longum* and *Bifidobacterium adolescentis*), is the most abundant form of short‐chain fatty acids. Acetate is the predominant SCFA species, contributing about 60% to the total SCFA pool in humans, with propionate and butyrate each contributing 20% (Topping and Clifton, 2001). Minor acids produced by gut microbiota include lactate isomers, valerate, and branched-chain SCFAs such as isobutyrate and iso-valerate (Topping, 1996).

These short-chain fatty acids are an important source of daily caloric intake. Acetate is notable among the SCFAs for its involvement in the regulation of satiation and body weight (Frost et al., 2014; González Hernández et al., 2019). Butyrate production is largely confined to Firmicutes, and it functions as the principal energy source for specialized epithelial cells of the large intestine and an important regulator of epithelial permeability and signaling pathways of inflammation. It is an important energy source for the brain, colon, and muscle cells (den Besten et al., 2013). Bacteroidetes are the most important producers of propionate, another common short‐chain fatty acid, although some Firmicutes are capable of production. It serves important functions in gluconeogenesis and cholesterol metabolism in the liver. Propionate has shown demonstrable capacity for hepatic gluconeogenesis (Yoshida et al., 2019). They are noted for their therapeutic potential, particularly butyrate, against diseases such as antibiotic-associated diarrhea, heart disease, colon cancer, and inflammatory bowel disease (Sharma and Shukla, 2016; Gill et al., 2018; Moss et al., 2018; Tominaga et al., 2018), and they also serve antimicrobial functions (Ciarlo et al., 2016).

### 4.2 Secondary bile acids

Primary bile acids (cholic and chenodeoxycholic) are products of cholesterol metabolism. They undergo microbial transformation in a two-step process (deconjugation and 7α/β‐dehydroxylation reactions) to produce secondary bile acids. First, the primary bile acid is deconjugated via enzymatic action of bile salt hydrolases (BSHs) from capable genera such as *Bifidobacterium*, *Lactobacillus*, and *Bacteroides*. Subsequently, enzymes secreted by bacteria belonging to *Clostridium* and *Eubacterium* catalyze the 7α/β-dehydroxylation (Busnelli et al., 2019; Chen et al., 2019). Following absorption of these SBAs in the colon, they can then be transported back to the liver to be conjugated (for recycling).

Through the modification of bile acids, gut microbiota can adjust the bodily composition of bile acids, modulating the various signaling pathways involved in metabolism and internal regulation. They are recognized as important signaling molecules in various processes, including energy, glucose, lipid, cholesterol, and drug metabolism, and are also potent immunomodulators (Li and Chiang, 2014; Chiang, 2013). These secondary acids serve as signaling molecules for receptors (nuclear and membrane) in the regulation of glucose and lipid metabolism, energy use, and epithelial permeability function.

### 4.3 Exopolysaccharides

Exopolysaccharides are secondary, high‐molecular‐weight carbohydrate polymers synthesized by many probiotic species, which can be attached to the cell or secreted into the environment. Several species of probiotic bacteria in various genera can produce large quantities of EPSs (Kodali et al., 2009; Indira et al., 2016; Maeda et al., 2004), particularly *Lactobacillus* and some *Bifidobacterium* strains. EPS can be divided into homopolysaccharides (one monosaccharide residue) and heteropolysaccharides (polymers with many different monosaccharide residues). The presence of amino sugars or glycerol in some has been reported (Badel et al., 2011). Their production is strain‐specific, although some probiotic strains can produce different types of EPS (Ibarburu et al., 2007; Puertas et al., 2018).

They possess various beneficial properties. They are known modulators of both innate and adaptive immune responses (Jin et al., 2010). They can act as stimulants for the release of interleukins and cytokines by macrophages for host defence (Ciszek-Lenda et al., 2013). They also act to enhance T-cell differentiation into CD3^+^, CD4^+^, and CD8^+^ cells (Matsuzaki et al., 2015). Through *in vitro* investigation, 0.1% of EPS of probiotic isolate *Lacticaseibacillus paracasei* M7 produced a hypolipidemic effect with a 70% reduction in total cholesterol compared with the control (Bhat and Bajaj, 2019).

*In vitro* studies, which form the bulk of the reported antioxidant potential of EPS, have been reported on oxygen radicals such as hydroxyl radical, superoxide radical, and DPPH (Tang et al., 2017). They show antimicrobial properties against a wide spectrum of opportunistic gut pathogens, including both Gram-positive and Gram-negative bacteria (Abdalla et al., 2021). They can be used as a substrate for increased production of bioactive SCFAs in the gut (Korakli et al., 2002; Salazar et al., 2009). It has been reported to possess inhibitory properties towards viruses (Al Kassaa et al., 2014; Kanmani et al., 2018). In addition, anticancer and antidiabetic properties have been reported (Koller et al., 2008; Huang et al., 2020).

### 4.4 Bacteriocins and antimicrobial peptides

Bacteriocins are peptides produced in the ribosomes by many probiotic strains (e.g., some *Lactobacillus*, *Bacillus*) with antimicrobial activity against closely related bacterial species and pathogenic strains. They are generally non-toxic and susceptible to the action of proteolytic enzymes, a feature that distinguishes them from many conventional antibiotics (Yang et al., 2014; Mokoena, 2017). They vary from heat-stable, low-molecular-weight molecules to larger ones that have undergone post-translational modifications. Through their inhibitory action, they eliminate pathogenic species and positively influence the competitive outcome for these probiotic species, shaping the microbial structure and reducing pro‐inflammatory triggers due to dysbiosis. They limit pathogenic colonization of the gut, helping probiotic species to be able to establish their niche for effective colonization. There is new evidence that their pathogen inhibition provides indirect metabolic benefits and helps enhance the microbial processes that improve host energy balance.

Probiotic organisms can synthesize several amino acids *de novo*. These amino acids can be used as precursors for the synthesis of SCFAs (Feng et al., 2018). The amino acids and their derivatives serve important regulatory functions in carbohydrate and lipid metabolism (den Besten et al., 2013). These amino acids can be used as substrates to produce equally bioactive molecules with important gut health functions. An important example is the aromatic amino acid tryptophan, which produces several important derivatives such as phenols and indole through probiotic fermentative action (Dai et al., 2015; Dodd et al., 2017). These amino acids serve important health roles for the host. D-tryptophan helps reduce hyperactivity in allergic reactions, functioning as an immunomodulator (Kepert et al., 2017). They also produce small molecules from amino acids that serve important roles in quorum sensing for biofilm formation. An example is S-adenosylmethionine, an intermediate in methionine metabolism, which can produce autoinducer peptides (Dai et al., 2011).

### 4.5 Tryptophan and indole derivatives

Tryptophan is an indole-based aromatic amino acid. *De Novo* synthesis is not possible in mammals and must therefore be acquired via dietary means. Many members of the gut microbiota, such as *Lactobacillus reuteri*, *Clostridium sporogenes*, *Escherichia coli*, and *Bifidobacterium infantis*, have the enzymatic capabilities for the conversion of unabsorbed tryptophan entering the colon into indole and indolic compounds such as indole-3-acetic acid (IAA), indole-3-propionic acid (IPA), and indole-3-aldehyde (IAld) (Roager and Licht, 2018). Microbial tryptophan metabolism is strongly linked to intestinal integrity. Indole helps strengthen intestinal barrier function and reduce inflammation (Bansal et al., 2010). ILA and IAld both serve important roles in immunomodulation (Xue et al., 2023). These indole derivatives can function as activators of receptors such as AhR or pregnane X receptor (PXR) (Lamas et al., 2018; Natividad et al., 2018; Li et al., 2024).

These metabolites can also contribute to mucosal integrity and pathogen suppression (Wlodarska et al., 2017; Zelante et al., 2013). Its colonization resistance potential was observed against *Candida albicans*. Increased indole concentration produced inhibitory effects on certain pathogenic species such as *Citrobacter rodentium* and enterohemorrhagic *Escherichia coli* via the suppression of the expression of their virulence factors (Kumar and Sperandio, 2019).

Indole and indolic compounds have a modulatory role on immune cells, cytokine and interleukin production, as they are endogenous AhR ligands (Bansal et al., 2010; Krishnan et al., 2018). ILA can regulate the differentiation of colonic macrophages to suppress colitis-associated tumorigenesis (Li et al., 2024). IPA and IAA have good antioxidant properties, with the capacity for free-radical scavenging (Dragicevic et al., 2011; Zhong et al., 2025a). IPA is known to mediate mitochondrial biogenesis and reduce oxidative damage (Liu et al., 2020; Gundu et al., 2024; Zeng et al., 2025), even enhancing the metabolic function of mitochondria in diseased metabolic states (Gesper et al., 2021). They are also important in regulating energy metabolism. The administration of IPA for four weeks ameliorated glucose metabolism (anti-diabetic potential) in a male Wistar rat model of high-fat diet/streptozotocin-induced diabetes (Shuja et al., 2026).

### 4.6 Conjugated linoleic acids

Through an isomerization reaction, certain probiotic strains can transform linoleic acid into conjugated linoleic acids. They are positional and geometric (*cis* or *trans*) isomers of linoleic acid containing a conjugated double bond. The most naturally occurring CLAs in most foods are rumenic acid (*cis* - 9, *trans* - 11) and *trans* - 10, *cis* - 12 isomers (de Carvalho et al., 2010). LAB are good producers of CLA isomers using LA as a precursor (Nasrollahzadeh et al., 2023; Terán et al., 2015). Among this group, *Lactiplantibacillus plantarum* is the best-known CLA producer (Kishino et al., 2002; Khan et al., 2023). CLAs have garnered significant scientific attention in recent times due to perceived health benefits.

Potential health benefits include immunomodulatory, anti-diabetic, anti-carcinogenic, anti-atherosclerotic, anti-osteoporotic, anti-obesity, and anti-inflammatory effects (Dachev et al., 2021; Malinska et al., 2015; Stachowska et al., 2012; DeGuire et al., 2012; Lehnen et al., 2015). It is also reported to lower LDL-cholesterol levels (Derakhshande-Rishehri et al., 2015). Available literature indicates a definite causal link between CLAs and these purported benefits. Mechanisms include stimulation of fatty acid oxidation, modification of adipocyte differentiation, and modulation of peroxisome proliferator‐activated receptors (PPARs). Bioactivity of CLAs is strain-specific and also shows variations with isomer composition.

### 4.7 Vitamins and cofactors

Humans require a dietary supply of vitamins because humans have mostly lost the capacity for *de novo* synthesis over time. Insufficient consumption of required vitamins can lead to malnutrition and stunted growth. They also improve the antioxidant system, methylation pathways, and hepatic and peripheral host metabolism. The probiotic contribution to overall host health quantitatively depends on microbial gut composition, dietary intake, and level and capacity for intestinal absorption. Water-soluble vitamins are, as expected, soluble in water, making their absorption in the intestine direct and easy, while fat-soluble ones must be absorbed through lipid micelles.

Many probiotic species are capable of biosynthesis of several B‐group vitamins, including riboflavin (B2), folate (B9), B12, and structural analogues of vitamin K. Natural producers of B group vitamins (B1, B2, B3, B6, B8, B9, B12) include *Lactobacillus plantarum*, *Lactobacillus reuteri*, *Bifidobacterium adolescentis*, and *Bifidobacterium pseudocatenulatum* (Pompei et al., 2007; Gu et al., 2015; Li and Gu, 2016). Folic acid is an important B-complex vitamin that can be produced copiously by some gut microbiota. It is important in amino acid conversion, nucleic acid synthesis, and antioxidant potential. Common folate-producing lactic acid bacteria include *Lactococcus lactis*, *Streptococcus thermophilus*, and *Bifidobacterium* spp. *L*. *plantarum* is a notable folate producer among the traditional lactobacilli, since most cannot synthesize it and require an exogenous supply of it. A probiotic species capable of producing cobalamin is *Limosilactobacillus reuteri* (Mohammed et al., 2014).

Treatment of tamoxifen-induced endometrial carcinoma in rats used a combination of B-complex vitamins (riboflavin and niacin) and vitamin C (ascorbic acid) (Sundravel et al., 2006). *Bifidobacteria* have also been observed to be significant producers of thiamine compared to LAB (Masuda et al., 2012).

### 4.8 Gamma‐aminobutyric acid and neuroactive metabolites

Through a decarboxylation reaction, several *Lactobacillus*, *Levilactobacillus*, *Lactiplantibacillus*, *Lacticaseibacillus*, and *Bifidobacterium* strains can convert glutamate into GABA. It serves as an important modulator in hormonal production, lipid oxidation, protein synthesis, and regulation of blood pressure (Behbahani et al., 2020). Increased production of GABA can be obtained via the inhibition of GABA aminotransferase (Cui et al., 2020). The neuroactive metabolites include serotonin precursors and catecholamine modulators. Together, they act as signaling molecules for the enteroendocrine pathway, vagal afferents, and immune mediators, modulating information flow from the gut to the brainstem. Therefore, they play important metabolic, stress, and appetite roles in the host.

## 5 Mechanisms of action of probiotic-derived metabolites in metabolic disorders

Generally, probiotic-derived bioactive metabolites can exert their beneficial potential using several mechanisms that are molecularly and cellularly interconnected. These mechanisms are indicated to influence intestinal function, immune responses, energy metabolism, oxidative balance, and gene regulation. The metabolites do not act independently but interact with host receptors, signaling pathways, and the resident microbiota to restore metabolic homeostasis and attenuate the progression of obesity, insulin resistance, type 2 diabetes mellitus (T2DM), metabolic dysfunction-associated steatotic liver disease (MASLD), and other metabolic disorders (Koh et al., 2016; Fan and Pedersen, 2021). Their major mechanisms of action include modulation of gut barrier integrity, regulation of host immune responses, modulation of glucose metabolism, lipid metabolism and energy homeostasis, modulation of the gut–brain axis, and antioxidant mechanisms.

### 5.1 Modulation of gut barrier integrity

The first line of defense separating luminal microorganisms from systemic circulation is the intestinal epithelial barrier. When this barrier becomes dysfunctional, it could lead to increased intestinal permeability (“leaky gut”), thus allowing translocation of microbial products such as lipopolysaccharide (LPS), which contributes to chronic low-grade inflammation and metabolic disorders (Cani, 2018).

The presence of probiotic-derived metabolites enhances epithelial tight junctions through an increase in the expression and assembly of Zonula occludens-1 (ZO-1), Occludin, Claudin-1, and Claudin-4. Some SCFAs, particularly butyrate, can activate AMP-activated protein kinase (AMPK), promote tight junction assembly, and epithelial repair (Peng et al., 2009). Also, butyrate can stimulate intestinal epithelial cell proliferation and differentiation and accelerate mucosal healing. In addition, indole derivatives that are produced from tryptophan metabolism can activate AhR, which can enhance epithelial regeneration and mucus production (Roager and Licht, 2018).

Metabolic endotoxemia results from increased translocation of bacterial lipopolysaccharide (LPS) into circulation. Probiotic metabolites reduce circulating LPS concentrations by strengthening epithelial integrity, stimulating mucin secretion, reducing pathogen colonization, and promoting beneficial microbiota. When there is reduced endotoxemia, there is decreased activation of Toll-like receptor 4 (TLR4), thereby suppressing systemic inflammation (Cani et al., 2007).

The primary source of energy for colonocytes is butyrate. The presence of SCFAs can stimulate mucus layer formation, goblet cell differentiation, and antimicrobial peptide secretion. Also, EPSs that are produced by *Lactobacillus*, *Lactiplantibacillus*, *Limosilactobacillus*, and *Bifidobacterium* are known to enhance epithelial barrier function by improving bacterial adhesion and biofilm stability (Salazar et al., 2016).

### 5.2 Regulation of host immune responses

Probiotic-derived metabolites regulate both innate and adaptive immune responses, which can help in the prevention of chronic inflammation associated with metabolic diseases. This is achieved through the stimulation of anti-inflammatory cytokines, NF-κB inhibition, and immune homeostasis. Several SCFAs are known to increase production of anti-inflammatory cytokines, including IL-10 and transforming growth factor-beta (TGF-β). They can also simultaneously suppress pro-inflammatory cytokines, such as TNF-α, IL-1β, IL-6 and IL-17. Differentiation of regulatory T cells (Tregs) and maintenance of immune tolerance are promoted by butyrate. Butyrate is also indicated to inhibit activation of NF-κB through HDAC inhibition, reduced IκB degradation, and suppression of inflammatory gene transcription. A reduction in NF-κB activity also lowers inflammatory mediator production in obesity and T2DM. Nuclear factor-kappa B (NF-κB) is a central regulator of inflammatory signaling (Furusawa et al., 2013).

Also, probiotic metabolites can regulate immune balance through dendritic cell maturation, macrophage polarization (M2 phenotype), expansion of regulatory T cells, and balance of Th1/Th2 responses. Indole metabolites activate AhR signaling and maintain mucosal immune tolerance (Zelante et al., 2013).

### 5.3 Modulation of glucose metabolism

Several probiotic metabolites have been implicated in the modulation of glucose metabolism directly through endocrine and metabolic signaling. When SCFAs activate GPR41 (FFAR3) and GPR43 (FFAR2), improvement of insulin sensitivity can occur. The presence of these receptors improves insulin signaling, glucose uptake, and metabolism in adipocytes. Butyrate can reduce insulin resistance through suppression of chronic inflammation and improvement of mitochondrial function (Canfora et al., 2015).

Also, SCFAs have been reported to stimulate enteroendocrine L cells and increase secretion of GLP-1 and peptide YY (PYY). This could result in improved insulin secretion, delayed gastric emptying, increased satiety, and reduced food intake. Propionate serves as a substrate for hepatic metabolism while simultaneously regulating glucose production. The presence of SCFAs suppresses excessive gluconeogenesis by activating AMPK and reducing expression of phosphoenolpyruvate carboxykinase (PEPCK) and glucose-6-phosphatase (G6Pase). It could also lead to improved hepatic insulin sensitivity, contributing to better glycemic control (Tolhurst et al., 2012).

### 5.4 Lipid metabolism and energy homeostasis

Probiotic metabolites regulate lipid metabolism through multiple metabolic signaling pathways. The oxidation of fatty acids, such as butyrate, activates AMPK and PPAR-α. This can result in increased β-oxidation, reduced hepatic lipid accumulation, and improved mitochondrial function (Blaut, 2015).

Short-chain fatty acids suppress expression of lipogenic genes, such as SREBP-1c, fatty acid synthase (FASN), and acetyl-CoA carboxylase (ACC). Reduced lipogenesis decreases hepatic steatosis and adipose tissue expansion.

In addition, AMPK activation promotes increased glucose uptake, enhanced fatty acid oxidation, inhibition of cholesterol synthesis, improved mitochondrial biogenesis, and reduced inflammatory signaling. Butyrate is among the strongest microbial activators of AMPK.

### 5.5 Modulation of the gut–brain axis

Gut microbial metabolites influence bidirectional communication between the gastrointestinal tract and central nervous system. This is achieved through neuroendocrine signaling or appetite and satiety regulation. Probiotic metabolites stimulate vagal afferent pathways, enteroendocrine signaling, and neurotransmitter synthesis. The important metabolites include GABA, serotonin precursors, dopamine precursors, and indole derivatives. These regulate mood, stress responses, and metabolic function. Furthermore, SCFAs increase secretion of GLP-1 and PYY. These hormones reduce appetite, delay gastric emptying, increase satiety, and improve energy balance. Acetate may also directly influence hypothalamic appetite centers (Cryan et al., 2019).

### 5.6 Antioxidant mechanisms

One major contributor to insulin resistance and metabolic dysfunction is oxidative stress. Several probiotic metabolites can reduce reactive oxygen species (ROS) through the scavenging of free radicals, reduction in lipid peroxidation, suppression of inflammatory oxidants, and improvement of mitochondrial function. *Lactobacillus*, *Limosilactobacillus*, *Lactiplantibacillus*, *Lacticaseibacillus*, strains can produce glutathione and antioxidant peptides.

In addition, SCFAs activate the *Nrf2* signaling pathway, increasing expression of superoxide dismutase (SOD), catalase (CAT), glutathione peroxidase (GPx), and heme oxygenase-1 (HO-1). The activation of these enzymes enhances cellular antioxidant defense (Tripathi et al., 2024).

### 5.7 Epigenetic and gene regulatory mechanisms

There is growing evidence that indicates probiotic-derived metabolites function as epigenetic regulators influencing host gene expression. Histone deacetylase (HDAC) inhibition and butyrate are potent endogenous inhibitors of HDACs. The inhibition of HDAC can promote histone acetylation and chromatin relaxation. It can also lead to transcription of anti-inflammatory genes and suppression of inflammatory mediators (Donohoe et al., 2012).

Probiotic metabolites regulate the expression of genes involved in glucose metabolism, lipid metabolism, inflammation, oxidative stress, and intestinal barrier integrity. The key signaling pathways include AMPK, NF-κB, Nrf2, PPARs, AhR, FXR and TGR5. Collectively, these molecular mechanisms contribute to improved metabolic homeostasis and reduced progression of obesity, insulin resistance, T2DM, and MASLD.

## 6 Therapeutic potential in specific metabolic disorders

### 6.1 Obesity

Acetate, propionate, and butyrate (short-chain fatty acids) have the most compelling evidence for the positive effects of probiotic-derived metabolites under obese conditions. They act as stimulants for enteroendocrine hormones and regulate energy homeostasis using G protein-coupled receptors in a process linked to their fermentative activity, and produce effects in tissues of the liver, muscles, and adipose (Wu et al., 2023; Fang et al., 2024; Cui et al., 2025; Li et al., 2025). A well-reported mechanism is the increased secretion of GLP-1 and PYY through the modulation effort of FFAR2/FFAR3 or GPR41/43, to regulate energy metabolism, reduce appetite, and reduce insulin resistance (Li et al., 2025; Zhong et al., 2025b). There is evidence for their ability to stimulate fatty acid oxidation over PPARγ-driven adipogenesis and hepatic metabolism by activating AMPK-related pathways (Nazarinejad et al., 2025; Fang et al., 2024).

The body of evidence also suggests the importance of factors such as site, route, and quantity on the eventual outcome of SCFA application, with oral, dietary intake producing a less effective and direct outcome on metabolic and endocrine parameters than colonic delivery (Fang et al., 2024). This scenario creates the apparent lack of consensus in the literature over the holistic and comprehensive effects of SCFA on obesity-related outcomes.

Another important strategy is the attenuation of metabolic endotoxemia. Dysbiosis due to obesity leads to the activation of TLR4–NF-κB signaling, which stimulates chronic inflammation and reduced insulin sensitivity, a situation caused by the increased channeling of LPS due to increased permeability of the intestinal epithelium (Cui et al., 2025). Probiotic-derived compounds such as SCFA-rich cell-free supernatants, dead cells, and subcellular components have shown intestinal tightening and anti-inflammatory effects (Nazarinejad et al., 2025).

Other important metabolites include secondary bile acids, although their effects are modulated by chemical nature or dose. Lithocholic acid can suppress lipogenesis and TGR5 signaling and increase energy use, while other secondary bile acids can promote adiposity and lipid storage (Fang et al., 2024; Cui et al., 2025). Indole-3-lactic acid, a tryptophan derivative, is associated with the promotion of the production of enteroendocrine cells through cellular differentiation of intestinal stem cells (Li et al., 2025). Other notable effects of indole derivatives include increased intestinal tightening, reduced adiposity and endotoxemia, as well as AhR activation and IL-22 signaling (Zhang et al., 2025).

The preclinical evidence using animal models is quite strong. Evidence from human trials seems to be variable and mixed. While improved anatomical and biochemical parameters, such as waist girth, insulin and triglyceride levels, and C-reactive protein, were reported in a 2025 meta-analysis of 25 randomized trials, overall reductions in BMI or body weight were not significant (Li et al., 2025). Overall, current knowledge seems to indicate that they produce positive effects on inflammation, fat distribution, and metabolic outcomes that are apparent in weight loss effects (Li et al., 2025). However, important and yet unanswered questions remain about dose and delivery site for effects beyond the modest ones typically recorded.

### 6.2 Type 2 diabetes mellitus

The mechanistic effects of probiotic-derived bioactive metabolites on Type 2 diabetes mellitus show modest effects rather than definitive, radical changes. These effects, which typically include reduced insulin resistance, inflammation, intestinal permeability, and improved incretin signaling, are typically mediated through the action of SCFAs and secondary bile acids (Zhang et al., 2021; Du et al., 2022; Karthick et al., 2026).

The fact that not all metabolites exhibit positive effects on T2DM is well established. Some, such as SCFAs, secondary bile acids, and tryptophan-derived metabolites, have modulatory effects on metabolic processes; on the other hand, trimethylamine N-oxide, imidazole, and highly branched amino acids tend to increase β-cell stress, inflammatory response, and insulin resistance (Wu et al., 2023; Zhang et al., 2021; Zhou et al., 2022; Mendis et al., 2025). Hence, the germane question is how to impact the right pathway to produce the desired glycemic benefits.

GPR41 and GPR43 signaling through SCFAs can increase the biosynthesis of GLP-1 and PYY while inhibiting inflammatory response (Karthick et al., 2026; Yu et al., 2025). Epigenetic and metabolic effects via microbial fermentation can be derived from butyrate-induced inhibition of histone deacetylase and AMPK/p38 activation (Karthick et al., 2026). Instructively, T2DM is typically linked to butyrate depletion and reduced SCFA activity (Yu et al., 2025). Notably, human trials and animal models have revealed the positive impact of enriching the supply of SCFAs in reducing inflammation and promoting insulin sensitivity (Karthick et al., 2026).

Positive benefits can also be derived from secondary bile acids. Their effects have been observed in both metabolic and inflammatory processes (Karthick et al., 2026). Recent evidence points to their functional role in regulating glucose homeostasis in animal models and human trials (Chen et al., 2023; Zhang et al., 2025). Higher indole propionate levels show correlations with a lower risk of diabetes, and other tryptophan derivatives have also shown anti-inflammatory effects (Mendis et al., 2025; Karthick et al., 2026). Characteristic features of T2DM-associated dysbiosis include damaged intestinal epithelial linings, mild endotoxemia, and chronic inflammation (Chen et al., 2023; Nikolaidis et al., 2025).

Current knowledge about the efficacy of probiotic-derived metabolites is limited and varied in strength of evidence at the clinical phases. Recent meta-analytic and systematic reviews show modest outcomes (Ayesha et al., 2023; Paquette et al., 2023; Nikolaidis et al., 2025). The current state of knowledge mandates that they be treated as adjunctive compounds rather than standard therapeutic agents (Nikolaidis et al., 2025; Panwar et al., 2013). While SCFAs and secondary bile acids have shown early clinical promise, there is still a need for standardized formulations and trials using selected and specific metabolites.

### 6.3 Metabolic syndrome

The core characteristics of metabolic syndrome (MetS) include insulin resistance, hypertension, dyslipidemia, oxidative stress, central adiposity, and chronic low-grade inflammation, all possible functional targets for metabolite interventions (Fabersani et al., 2021; Li et al., 2021; Maurya et al., 2025).

The body of evidence strongly supports the preclinical efficacy of SCFAs over other metabolites. For metabolic syndrome, their noted effects include improved insulin sensitivity, reduced inflammation, adipogenesis, plasma cholesterol levels, and GLP-1 and GLP-2 secretion (He and Shi, 2017; Hu et al., 2018; Yu et al., 2025). They can also have an ameliorative effect on cytokine production linked to inflammation, a distinct feature of endotoxemia (Zhang et al., 2023; Kopczyńska & Kowalczyk, 2024).

The impact of other metabolites is less clear. It is thought that secondary bile acids have a role to play in MetS via insulin sensitivity, cholesterol metabolism, and lipid handling (He & Shi, 2017; Olalekan et al., 2024). Tryptophan derivatives seem to function through immunomodulatory roles, signaling via the aryl hydrocarbon receptor (Scott et al., 2020), and adipose tissue metabolism (Olalekan et al., 2024). Clinical trials suggest positive but no confirmatory evidence for these metabolites, as shown by their beneficial effects on LDL-cholesterol, fasting blood glucose, total cholesterol, triglycerides, insulin, and body fat percentage (Dong et al., 2019; Tenorio-Jiménez et al., 2020; Chen et al., 2023).

## 7 Experimental and clinical evidence

A growing body of experimental and clinical evidence supports the role of gut microbiota- and probiotic-derived metabolites in improving metabolic health (Fan and Pedersen, 2021).

Results from *in vitro* studies, animal experiments, and human clinical trials have consistently revealed that probiotic metabolites exert beneficial effects through several complementary mechanisms. Most probiotic metabolites are indicated to modulate inflammatory responses, enhance intestinal barrier integrity, improve glucose metabolism and insulin sensitivity, regulate lipid metabolism and energy homeostasis, reduce oxidative stress, and modulate host immune responses (Markowiak-Kopeć and Śliżewska, 2020).

*In-vitro* studies have provided valuable mechanistic insights into the molecular and cellular pathways through which these metabolites exert their biological effects. Findings from animal models have shown that these mechanisms under physiological conditions demonstrate improvements in metabolic outcomes such as obesity, insulin resistance, hepatic steatosis, and chronic inflammation. In addition, some human clinical trials have begun to establish the therapeutic efficacy, safety, and clinical applicability of these metabolites in the prevention and management of metabolic disorders. Collectively, evidence from these complementary research approaches provides a strong scientific foundation for the development of these metabolites as novel therapeutic agents for metabolic diseases (Agus et al., 2021; Fan and Pedersen, 2021).

### 7.1 Translational considerations: dose, delivery and pharmacokinetics

Direct administration of microbial metabolites presents an important translational challenge because their biological effects depend not only on dose but also on chemical stability, absorption, distribution, metabolism, elimination, and exposure at the intended site of action. This is particularly relevant for SCFAs, for which the route and site of delivery can influence their metabolic and endocrine effects, with colonic delivery potentially producing responses that differ from those achieved through conventional oral or dietary administration (Fang et al., 2024). Consequently, strategies aimed at achieving controlled and site-specific metabolite exposure warrant greater attention. Potential approaches include encapsulation, enteric-coated formulations, polymer-based controlled-release systems, targeted colonic delivery, and the use of metabolite-producing microbial or synbiotic approaches. Such strategies may help to protect bioactive compounds from premature degradation or absorption and facilitate more sustained exposure at physiologically relevant concentrations. Future studies should therefore establish pharmacokinetic–pharmacodynamic relationships, including absorption, distribution, metabolism, excretion, tissue exposure, and concentration–response relationships, before optimal therapeutic doses and delivery regimens can be reliably established (Fang et al., 2024).

### 7.2 *In-vitro* studies

Studies using *in-vitro* models have been vital in understanding the molecular mechanisms through which these metabolites influence host metabolism. These studies provide precise investigation of individual metabolites under controlled experimental conditions without the complexity of whole-organism interactions. Cell culture models involving human intestinal epithelial cell lines, including Caco-2, HT-29, and T84, are commonly used to investigate the effects of metabolites on intestinal barrier function (Plaza-Díaz et al., 2019).

Some SCFAs, such as butyrate, are known to significantly increase the expression of tight junction proteins such as ZO-1, occludin, and claudin-1, thereby enhancing epithelial barrier integrity and reducing intestinal permeability (Peng et al., 2009). In addition, the presence of these metabolites could stimulate mucin production and improve differentiation of epithelial cells, which could help in strengthening the first line of defense against microbial translocation. In some studies that used macrophage (RAW264.7) and monocyte (THP-1) cell lines, it is reported that butyrate, propionate, and indole derivatives can suppress the production of pro-inflammatory cytokines including TNF-α, IL-6, and IL-1β while also increasing anti-inflammatory cytokines such as IL-10 (Vinolo et al., 2011).

Furthermore, studies on hepatic cell lines (e.g., HepG2) have revealed that SCFAs can lead to a reduction in intracellular lipid accumulation by suppressing lipogenic genes and activating AMPK, which is an indication of potential benefits against metabolic dysfunction-associated steatotic liver disease (MASLD). Also, studies in adipocyte models (3T3-L1 cells) have reported that the presence of these metabolites could lead to inhibition of adipocyte differentiation and enhancement of insulin signaling through an increase in glucose transporter type 4 (GLUT4) expression and reduction of inflammatory signaling.

Several molecular pathways targeted by these metabolites have been identified using mechanistic studies. This is carried out through the activation of G-protein-coupled receptors [such as free fatty acid receptor 2 (FFAR2/GPR43) and free fatty acid receptor 3 (FFAR3/GPR41)] and activation of AMPK signaling, leading to enhanced glucose uptake, increased fatty acid oxidation, and reduced lipogenesis. Also, metabolites, such as butyrate, are known to inhibit histone deacetylases and suppress NF-κB activation, which can lead to decreased production of inflammatory mediators. Similarly, indole derivatives are reported to activate AhR, which is known to promote intestinal barrier integrity and immune regulation. Some other metabolites have been implicated in the stimulation of the nuclear factor erythroid 2-related factor 2 (Nrf2) pathway, increasing cellular antioxidant defenses and reducing oxidative stress (Donohoe et al., 2012).

### 7.3 Animal models

Findings from animal studies provide valuable information regarding the physiological effects of these metabolites and bridge the gap between mechanistic investigations and clinical application. In rodent models, most investigations have employed mouse and rat models of obesity, diabetes, metabolic syndrome, and MASLD induced by high-fat diets or genetic manipulation. Supplementation with butyrate consistently improves insulin sensitivity, glucose tolerance, body weight regulation, lipid metabolism, and intestinal barrier integrity (Gao et al., 2009).

Evidence has shown that administration of propionate reduces hepatic lipid accumulation and improves energy expenditure by activating intestinal gluconeogenesis. Also, supplementation with acetate has been associated with improved appetite regulation through enhanced secretion of GLP-1 and PYY. In addition, indole derivatives improve intestinal barrier integrity, reduce systemic inflammation, and protect against obesity-induced insulin resistance. Secondary bile acids generated by probiotic metabolism are known to activate FXR and TGR5, thereby improving glucose and lipid metabolism. Several probiotic strains capable of producing these metabolites, such as *Lacticaseibacillus rhamnosus*, *Lactiplantibacillus plantarum*, *Bifidobacterium longum*, and *Akkermansia muciniphila* have demonstrated beneficial metabolic effects in rodent models (Fan and Pedersen, 2021).

Animal studies suggest that these metabolites exert multiple complementary effects that collectively improve metabolic health. These include restoration of intestinal barrier integrity, reduction of metabolic endotoxemia, suppression of chronic low-grade inflammation, improved insulin sensitivity, enhanced mitochondrial function, reduced hepatic steatosis, and improved lipid oxidation (Agus et al., 2021). Some of the translational limitations of animal models have been reported. Rodent gut microbiota differs considerably from humans. Also, experimental doses of physiologically achievable concentrations and controlled laboratory environments may not accurately reflect the complexity of human dietary and lifestyle factors. In addition, data on long-term safety remain limited. However, despite these limitations, animal studies provide strong biological plausibility supporting clinical investigation (Nguyen et al., 2015; Walter et al., 2020).

### 7.4 Human clinical trials

Human intervention studies have increasingly evaluated the efficacy of probiotics capable of producing beneficial metabolites or directly assessed the effects of microbial metabolites themselves. Clinical trials have reported improvements in several metabolic parameters following probiotic supplementation.

Some of the reported benefits include improved insulin sensitivity, reduced fasting blood glucose, decreased HbA1c, lower serum triglycerides, reduced total cholesterol, decreased inflammatory biomarkers, and improved intestinal barrier function. Studies and meta-analyses indicate that probiotic supplementation produces modest but statistically significant improvements in glycemic control among patients with type 2 diabetes mellitus (Yao et al., 2017). To assess the therapeutic efficacy, several mechanisms likely contribute to the observed clinical benefits. Some of the benefits include increased SCFA production, improved gut microbial diversity, reduced intestinal permeability, decreased circulating endotoxin levels, enhanced GLP-1 secretion and reduced systemic inflammation (Agus et al., 2021).

In terms of safety profiles, current evidence indicates that probiotics and their metabolites are generally safe for healthy individuals and patients with metabolic disorders. The reported safety findings include excellent gastrointestinal tolerance, low incidence of adverse effects, absence of serious toxicity in most randomized controlled trials, and high compliance during prolonged supplementation. It is, however, advised that caution should be taken when administering to immunocompromised patients, critically ill individuals, and patients with severe intestinal barrier dysfunction. Long-term safety studies remain necessary, particularly for next-generation probiotics and purified postbiotic formulations (Salminen et al., 2021; Zhang et al., 2005).

## 8 Conclusion

Gut microbiota and probiotic-derived metabolites are indicated to provide functional benefits in host metabolic health. Their effects extend beyond the direct presence or colonization of live probiotic microorganisms. Several probiotic-derived metabolites, such as SCFAs, secondary bile acids, tryptophan and indole derivatives, EPSs, bacteriocins, conjugated fatty acids, vitamins, and neuroactive compounds, are indicated to contribute to the regulation of intestinal and systemic physiological processes. Extensive *in-vitro* and animal models provide strong mechanistic evidence that supports their roles in maintenance of intestinal barrier integrity, improvement of insulin sensitivity, regulation of lipid metabolism, appetite control, reduction of inflammation, and promotion of metabolic homeostasis.

Human trials increasingly demonstrate improvements in selected metabolic, inflammatory, and biochemical outcomes. Although clinical evidence remains encouraging, it is still relatively insufficient to establish metabolites as replacements for established therapies, with human studies reported to show modest and variable improvements in glycemic control, lipid profiles, inflammatory markers, insulin sensitivity, and other metabolic parameters.

A comparative analysis of live probiotics, postbiotics, and purified microbial metabolites reveals that metabolites may offer important advantages, such as greater stability, more precise dosing, reduced concerns regarding colonization, and potentially improved safety and formulation characteristics. These properties make them attractive candidates for the development of next-generation metabolic therapeutics. However, further translational research is required to address constraining issues surrounding metabolite composition, dosage, route of administration, and host characteristics such as age, sex, diet, and gut microbiota. A proposed structured future-research roadmap for translational results should include these priority areas (**Figure 1**). Such factors may partly explain the inconsistent outcomes observed among clinical studies; there is, however, a need for safety and long-term efficacy studies, particularly for concentrated or purified postbiotic and metabolite preparations and interventions intended for prolonged use or administration to vulnerable populations.

**Figure 1:**
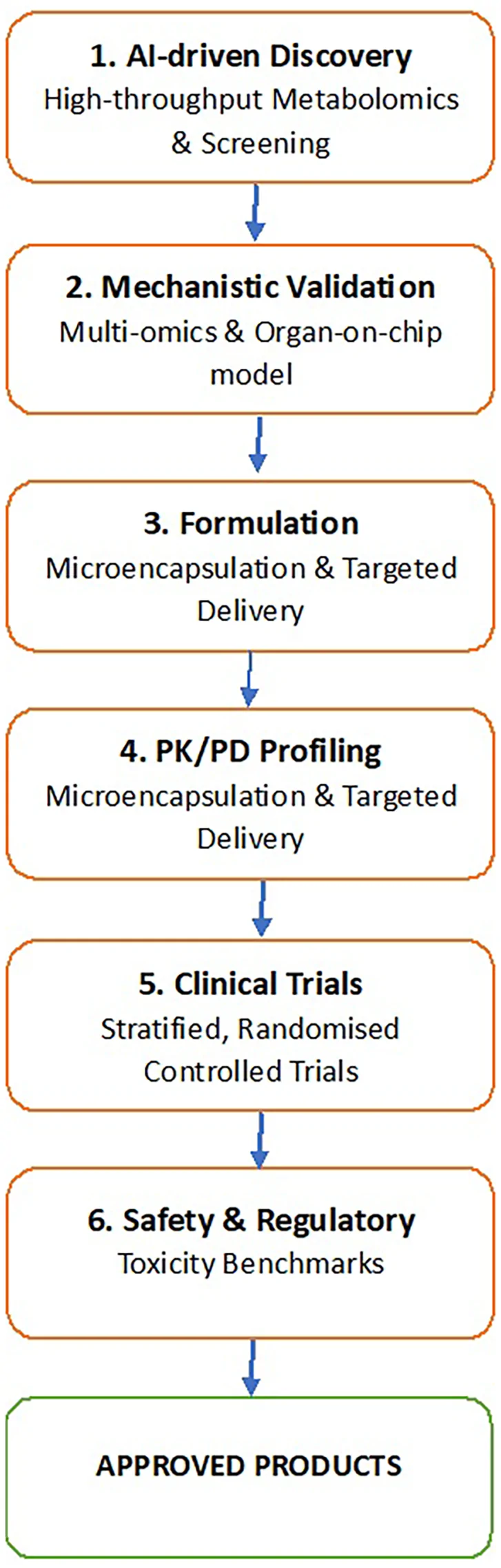
Future-research roadmap for gut microbiota-derived metabolite, and vice versa.

Overall, gut microbiota- and probiotic-derived metabolites constitute a promising and rapidly developing therapeutic frontier in metabolic disease management. Although substantial translational challenges remain, the strong prevailing preclinical evidence supports continued investigation of these microbial products as potentially complementary approaches to the prevention and management of metabolic disorders.
